# Determinants of Default in P2P Lending

**DOI:** 10.1371/journal.pone.0139427

**Published:** 2015-10-01

**Authors:** Carlos Serrano-Cinca, Begoña Gutiérrez-Nieto, Luz López-Palacios

**Affiliations:** Department of Accounting and Finance, University of Zaragoza, Zaragoza, Spain; Uppsala University, SWEDEN

## Abstract

This paper studies P2P lending and the factors explaining loan default. This is an important issue because in P2P lending individual investors bear the credit risk, instead of financial institutions, which are experts in dealing with this risk. P2P lenders suffer a severe problem of information asymmetry, because they are at a disadvantage facing the borrower. For this reason, P2P lending sites provide potential lenders with information about borrowers and their loan purpose. They also assign a grade to each loan. The empirical study is based on loans’ data collected from Lending Club (N = 24,449) from 2008 to 2014 that are first analyzed by using univariate means tests and survival analysis. Factors explaining default are loan purpose, annual income, current housing situation, credit history and indebtedness. Secondly, a logistic regression model is developed to predict defaults. The grade assigned by the P2P lending site is the most predictive factor of default, but the accuracy of the model is improved by adding other information, especially the borrower’s debt level.

## Introduction

Peer-to-peer (P2P) lending consists in individuals lending money to other individuals, without the intermediation of a financial institution. P2P can be analyzed under several approaches. It can be considered as an example of financial disintermediation [1], [2]; as another technological disruption provoked by Internet [3]; as a case of collaborative economy [4], or even as a platform to give loans to financially excluded people [5]. Although no traditional bank is present in the process, there is an electronic lending platform that mediates between borrowers and lenders of loans, charging a fee for this service [6]. Companies such as Prosper or Lending Club channel loans between individuals, whereas Kiva is focused on funding low-income people. P2P growth is remarkable, both in the number of loans and the number of investors, attracted by high returns expectations or socially responsible investment concerns [7], [8], [9].

The first research question of this paper aims at analyzing factors explaining default in P2P lending. P2P lending companies provide information on borrowers’ characteristics and loan purpose. Hence, each loan is rated with a grade that tries to capture the risk of default and thus investors can make their choices. If the P2P lending site does its job well; the lower the grade, the higher the default risk is and, consequently, the higher the interest rate will be. This paper analyzes the relationship among the grade, the interest rate and the default, empirically. It also poses a series of hypotheses on the relationship between default and the information provided by P2P lending companies on aspects such as loan size, loan purpose and borrower’s characteristics like annual income, indebtedness and credit history. The aim is to study the relevance of the information provided by the P2P lending site for lenders’ decision making and for lowering information asymmetry. In other words, if lenders should be only focused on interest rates or whether they should analyze additional factors. The empirical study uses data from Lending Club, the biggest US P2P lending company. The sample analyzed contains 24,449 loans. Although there is available information on all the funded loans from 2008 to 2014, only loans funded until 2011 can be analyzed, because the status of later loans (defaulted or non-defaulted) is still unknown. This happens because the minimum maturity of Lending Club loans is 36 months. For example, the status of a loan funded in September 2012 with 36 months maturity, cannot be known until September 2015. Hypotheses have been tested by using univariate means tests and survival analysis.

It is not only interesting to know factors explaining P2P loan default, but also to accurately predict loan defaults. The second research question presents a mathematical model to assess the predictive capability of the factors analyzed. There are several statistical techniques for credit scoring and default prediction, such as discriminant analysis, logistic regression, neural networks or classification trees, among others. Logistic regression is the most widespread technique, because it combines a high predictive capability with accuracy percentages not statistically significant different from other more recent techniques [10]. Classification techniques assign a 0 to defaulted loans and a 1 to non-defaulted loans. Explanation requires only cross validation whereas prediction requires intertemporal validation [11]. To do so, a primary sample is needed, called train sample, and to validate results, a test or holdout sample. The best outcome would be that the test sample will be gathered at a later time than the train sample, to ensure intertemporal validation. This has been done in this paper.

To the best of our knowledge, this is the first study explaining defaults in the Lending Club platform, using a database large enough to extract a holdout sample. Until recently, this was not possible due to data availability on the loan status. Our results show that, the higher the interest rate, the higher the probability of default is. The grade assigned by the P2P lending company is the best default predictor. Loan characteristics such as loan purpose; borrower characteristics like annual income, current housing situation, credit history and borrower indebtedness are related to default. However, other common drivers in default studies, such as loan amount or length of employment, have not a significant relationship with default within the data analyzed.

The remainder of the paper is organized as follows. Section 2 presents a related theoretical and empirical literature review on P2P lending. Section 3 presents the hypothesis development. Section 4 presents the data and the empirical results. Finally, conclusions are presented.

## Literature Review

P2P lending is a type of marketplace that connects the supply and demand of money through the Internet. Bachmann et al. [12] and Berger and Gleisner [6] review the history of P2P. It can be questioned whether it will become a disruptive innovation, as defined by [13], but it is clear that P2P lending is quickly spreading globally [14]. LendingClub issued $3.5 billion in loans in 2014, an important figure that nearly doubles the $1.9 billion of the previous year. But it is still far away from the data of any traditional bank, and it represents a small percentage compared to the $3.3 trillion in US consumer debt outstanding reported by the US Federal Reserve System in 2014 (see http://www.federalreserve.gov/releases/g19/current/).

Financial intermediation theory justifies P2P growth [7], [8], [9]. Financial intermediation is firstly explained by transactions costs [15]. Both conventional financial institutions and P2P lending bear customer evaluation costs before the loan is approved. Once the loan is approved, they also carry costs involved in monitoring loan payment as well as loan recovery costs [16]. However, P2P lending can lower other intermediation costs. Since it does not collect deposits, P2P lending is not subject to bank capital requirements, neither does it bear the Federal Deposit Insurance Corporation (FDIC) fee, and it is not overseen by bank regulators so far. P2P loans are not accounted on the books of the P2P lending platform, so no liability for the loans is needed. It does not experience financial frictions due to the coexistence of long term loans and short term deposits. Finally, although the use of Internet is not only for P2P lending, but also for online banking, automation reduces manual processes that would otherwise increase efficiency. Operating cost is the most important factor explaining interest margins in banking [17] and banks pass on their operating costs to their depositors and lenders [18]. This low intermediation costs could be transferred to clients in the form of higher revenues for lenders and lower interest rates for borrowers, compared to conventional financial institutions.

P2P lending sites also offer solutions to other formal credit market problem, credit rationing, which can explain their growth [19]. Market equilibrium equals supply and demand; if prices work, credit rationing should not exist, but it does exist [20]. Credit rationing means that some loan applicants may not receive a loan, even if they are willing to pay a high interest rate [20]. Credit rationing increases considerably in economic downturns [21]. Dehejia, Montgomery and Morduch [22] argue that financially excluded people seek access to credit, despite having to pay a high price. There are even socially responsible P2P platforms, where borrowers can obtain a loan to be reimbursed without paying interests; here, lenders are socially responsible investors. For example, Kivazip.org facilitates loans at 0% interest rate directly to entrepreneurs via mobile payments. But most financial entities try to follow the Pareto’s 80/20 principle when giving loans. More precisely, Hales [23] found that only 15% of all financial entities customers were profitable; in fact, fewer than 10% of bank’s clients produce 90% of its profits. Management manuals report similar figures [24]. There is a fat tail, with the best clients, served by private banking, and, in the other extreme, there is a long tail of small loans, served by microfinance. A priori, this is the less profitable part of the business because the fixed costs of dealing with small loans. Customer Relationship Management (CRM) systems are a practical implementation of Pareto’s principle in banks [25]. By using CRMs, banks group clients into several categories: from highly profitable to dispensable customers. Emekter, Tu, Jirasakuldech and Lu [26], by analyzing credit risk in P2P lending, find that borrowers with higher incomes and potentially higher scores do not participate in these markets. P2P operates in the long tail of small size loans. There are two strategies to obtain profits in the long tail. The first one is based on high interest rates, following the practices of microfinance institutions or even informal lending [27]. The second one is based on a high volume of small loans (high turnover strategy), which, in this context, implies applying technologies in an efficient way [27]. P2P lending tries to keep reasonable interest rates, following a high turnover strategy, by applying successful business models of some Internet companies that also operate in the long tail [28].

P2P lending is a risky activity for individual lenders, because the loans are granted by them, instead of P2P companies, which transfer the credit risk. Credit risk can be defined as the potential financial impact of any real or perceived change in borrowers’ creditworthiness, while creditworthiness is the borrowers’ willingness and ability to repay [29]. A credit score is a number that represents an assessment of the creditworthiness of a person, or the likelihood that the person will repay his or her debts [30]. P2P loans lack collateral or any kind of guarantee fund. So far, those interested in knowing the factors explaining loan default were risk analysts in financial institutions, specialized in avoiding, transferring or reducing risk. But the growing popularity of P2P is attracting individual investors who allocate part of their savings to personal loans, what is called P2P investing. Some of them lack enough knowledge on credit risk. P2P investing is not allowed in many countries and in some US states. Zeng [31] reviews and compares some of the legal aspects of P2P in different countries.

Transactions costs and credit rationing could explain P2P lending growth, but these entities face a fundamental problem: information asymmetry. Asymmetric information arises because borrowers are better informed than lenders of their ability and willingness to repay. In consequence, lenders are at a disadvantage. This is one of the main concerns in credit markets [20]. Leland and Pyle [32] Campbell and Kracaw [33] and Myers and Majluf [34] suggest that informational asymmetries may be a primary reason to explain financial institutions’ existence. It is not easy for an individual lender to distinguish borrowers with a high probability of default from solvent ones. In consequence, a risk expert is needed and this would justify the existence of banks. The bank, at least, has historical information on its clients, or even knows them personally; whereas an individual P2P lender, screening on his computer, hardly gets a profile with some borrower’s data. Information asymmetry leads to adverse selection, where lenders cannot discriminate between borrowers with different credit risks [35]. Adverse selection may be mitigated with quality information. If P2P lending companies just put lenders and borrowers into contact with each other, the information asymmetry problem would imply that few lenders would join the P2P credit market, and these companies would have disappeared by the lack of lenders. But P2P lending sites offer information on loan quality. While disintermediation is a primary characteristic of online P2P lending, these companies are in partnership with credit rating agencies to reduce the information asymmetry problem [1]. Miller [36] empirically finds that providing more information improves lender screening and dramatically reduces the default rate for high-risk loans, but has little effect on low-risk loans. P2P lending sites make an effort towards transparency in their lending process. They do not only provide detailed public information about each available loan, but they also allow downloading of historical information with all the loans funded, their characteristics and their status of being solvent or failed (for example, see Lendingclub.com: https://www.lendingclub.com/info/download-data.action; Prosper.com: https://www.prosper.com/tools/DataExport.aspx or Kiva.org: http://build.kiva.org/docs/data/). This contrasts with common traditional bank practices.

In the last years a number of empirical studies have been made using data from P2P lending platforms. Ruiqiong and Junwen [14] perform a recent revision on empirical research. Factors explaining successful funding of loans is a widely researched topic [1], [5], [19], [37], [38], [39]. Lin, Prabhala and Viswanathan [19] study if borrowers’ online friendships increase the probability of successful funding and its role in lowering ex post default rates. But they do not analyze the predictive capability or the accuracy of the model. Emekter, Tu, Jirasakuldech and Lu [26] evaluate the credit risk of P2P online loans, using Lending Club data, but they do not provide the model’s accuracy. Gonzalez and Loureiro [37] study the impact of borrower profiles, focusing on borrowers’ photographs and their results support the ‘beauty premium’ effect. Weiss, Pelger and Horsch [38] study credit bid’s funding success, with similar results. They also study the factors explaining loan final interest rate. They study P2P loan bidding and find that the most important factor lenders use to allocate funds is the rating assigned by the P2P lending site. Traditional banks rely on risk analysts who approve hundreds of operations. By contrast, P2P borrowers and lenders are involved in a social network [5]. Lenders themselves analyze and select borrowers. Lee and Lee [1] and Zhang and Liu [39] analyze lenders behavior in P2P lending, finding strong evidence of herding behavior among lenders.

## Hypothesis Development

It has been shown previously that it is important to study the relevance of the information provided by the P2P lending site for lowering information asymmetry, identifying the factors explaining P2P defaults. P2P lending platforms assign a grade to each loan, relying on third party information, like FICO score, used by the vast majority of banks and credit grantors. This grade is associated with an interest rate, depending on its credit risk. If P2P lending companies are accurate, high risk loans will be assigned with low grades and will be charged with high interest rates. Credit risk stems from the possibility of the borrower defaulting principal or interest payments, because of the inability or lack of willingness to pay them back. Being a risky investment, the lenders ask for a premium over the risk-free interest rate. The value of the credit spread over the risk-free interest rate is linked to credit quality, defined as the estimated default probability and the estimated loss in the event of default [40].

Interest rates should be more a matter of credit risk than a matter of cost [41]. There are several models to explain credit risk [42]. In the structural model by Merton [43] the structure of borrower’s liabilities, jointly with the fluctuations in the assets value, determines the probability of default and its payoff. Reduced models, such as Jarrow [44], are characterized by two assumptions: firstly, an exogenously given process for the loan’s default time; and secondly, an exogenously given process for recovery in case of default. Default probabilities are a random variable depending on interest rates and a risk factor. These models are useful for estimating default probabilities [45]. Therefore:

H1. The relationship between interest rate and risk of default in P2P is positive.

The fulfilment of Hypothesis 1 means that P2P lending companies contribute effectively to lower information asymmetries between borrowers and lenders. Hypothesis 2 studies the drivers of default in depth. A number of theoretical models explaining drivers of default for consumer credit have been developed, for example De Andrade and Thomas [46] and Durkin and Elliehausen [47]. These models are inspired by corporate bankruptcy models, by replacing the value of a firm’s assets by borrower characteristics as proxies of individual’s creditworthiness. De Andrade and Thomas [46] propose a credit risk model using option theory and the value of the borrower’s reputation. However, most credit scoring models have an empirical nature [30], [48], [49]. Moro, Cortez and Rita [49] analyze recent literature in business intelligence applications for the banking industry, finding that credit scoring is the main application trend. Altman, Resti and Sironi [50] affirm that credit-scoring prediction models are often only tenuously linked to an underlying theoretical model. Abdou and Pointon [51], in a review of 214 studies of credit scoring, detail the explicative factors most widely used in empirical studies. Thomas [52] also surveys credit scoring evaluations by conventional banks, and the variables used to evaluate the applicant capacity to reimburse the loan principal and interest payments. Two approaches exist in credit scoring: statistical and judgmental [53]. The statistical approach, by using data on past loans, provides the probability of default [54]. The judgmental approach is based on expertise of credit analysts [52]. This approach is useful when there is a lack of enough data to develop a statistical credit score. Hence, financial institutions rely on it, by using the knowledge of their financial experts [55]. However, some of the judgmental approaches used for particular lenders in P2P loan allocation lack rigor, being based on aspects such as beauty or attractiveness of borrowers [37].

Loan purpose is considered as one of the factors explaining the probability of default [56]. A loan to finance a car has not the same risk than a loan for starting a business. Cader and Leatherman [57] found that more than 40% of the firms did not survive after 3 years, using a sample of 90,134 observations. Knaup and Piazza [58] found that about 40% of the firms survived after 5 years, using data from the US Census and Employment. Phillips and Kirchhoff [59] found that three out of five new businesses close in the first five years. By contrast, the percentage of defaulted car loans is 3.59% according to Agarwal, Ambrose and Chomsisengphet [60], using a sample of 6,996 loans in different countries. This percentage is 0.88% in May 2015 in the USA, according to S&P/Experian Auto Default Indices.

Another factor is loan size. The relationship between risk and loan size has been largely discussed [30], [61], [62], [63]. There are arguments saying that risk grows when loan size lowers, but it also grows using the opposite arguments. Empirical studies show ambiguous results, with none of them being significant [61], [62]. Jiménez and Saurina [62], studying more than three million loans, find a negative relationship between risk and loan size, explained because institutions study large loans more carefully. But the larger the loan analyzed, the higher the probability of default is, for a given size of the borrower. What matters is not only the size of the loan [63], but also the repayment capability of the borrower [30] and the loss given default, that is, the share of a loan that is lost when a borrower defaults [63].

Credit scoring mathematical models usually include borrower characteristics, widely applied by bankers to reach a subjective judgment, what Altman, Resti and Sironi [50] call the 4 ‘Cs’ of credit: borrower character (reputation), capital (leverage), capacity (volatility of earnings) and collateral. The variables used in empirical studies include the length of time that workers have been with their current employer, current housing situation, borrower’s income and indebtedness ratios [64], [65]. Indebtedness relates debt or loan payments to income; and its relationship with solvency has been found relevant in both studies on corporate finance [43], [66] and consumer finance [46]. Given the empirical nature of these studies, some variables can exhibit a high discriminatory power in some studies, whereas in others they do not. An example is the study by Bravo, Maldonado and Weber [67], on Chilean micro-entrepreneurs’ loans, where income is not a relevant variable to predict default.

Credit history is another key issue in consumer credit scoring [52]. Even for small businesses, the owner’s credit history predicts defaults better than financial variables from annual statements do [68]. Asch [69] describes the method followed to obtain FICO ratings, those most widely used by the consumer credit industry, such as credit cards or even some P2P lending sites. Credit history is one of the key determinants in FICO ratings which includes variables such as payment history information on specific types of accounts (credit cards, retail accounts or mortgage), amounts owed, length of credit history, past-due incidences of delinquency in the borrower’s credit file, the number of derogatory public records, or the number of inquiries by creditors, amongst others.

P2P lending is just another way of providing loans. It is expected that the factors that usually predict loan default, such as loan and borrower characteristics, are also related to the risk of default in P2P lending. Therefore,

H2a. Loan characteristics, such as loan purpose and loan amount, are related to the probability of default in P2P lending.

H2b. Borrower characteristics, such as current housing situation, annual income, and employment length are related to the probability of default in P2P lending.

H2c. Credit history, a record of a consumer’s ability to repay debts, is related to the probability of default in P2P lending.

H2d. Personal indebtedness is related to the probability of default in P2P lending.

## Empirical Study

The sample used contains all the loans funded by Lending Club from January 2008 to September 2014. Lending Club is the biggest US P2P lending site, and the first in issuing an IPO in the New York Stock Exchange, in December 2014, being LC its symbol. A subsample has been extracted, containing funded loans whose status (defaulted or non-defaulted) is known: they are 24,449 loans of the period 2008–2011 (the data are available in https://www.lendingclub.com/info/download-data.action). Loans of the year 2007 have been removed, because they used different borrower information. 36 month loans have been selected, and 60 month loans have been excluded, since most of them are still outstanding loans. Loan status information for 36 months loans funded in 2012 will be available in 2015. Table 1 shows the variables of the study.

**Table 1:** Variables used in the study.

Variable	Definition
*Borrower Assessment*	
Grade	Lending Club categorizes borrowers into seven different loan grades from A down to G, A-grade being the safest
Subgrade	There are 35 loan subgrades in total for borrowers from A1 down to G5, A1-subgrade being the safest
Interest Rate	Interest rate on the loan
*Loan Characteristics*	
Loan Purpose	14 loan purposes: wedding, credit card, car loan, major purchase, home improvement, debt consolidation, house, vacation, medical, moving, renewable energy, educational, small business, and other
Loan Amount	The listed amount of the loan applied for by the borrower
*Borrower Characteristics*	
Annual Income	The annual income provided by the borrower during registration
Housing Situation	Own, rent and mortgage
Employment Length	The length of time (years) that workers have been with their current employer
*Credit History*	
Credit History Length	Number of days of credit history considering the date when the borrower’s earliest reported credit line was opened
Delinquency 2 Years	The number of 30+ days past-due incidences of delinquency in the borrower's credit file for the past 2 years
Inquiries Last 6 Months	The number of inquiries by creditors during the past 6 months
Public Records	Number of derogatory public records
Revolving Utilization	Revolving line utilization rate, or the amount of credit the borrower is using relative to all available revolving credit.
Open Accounts	The number of open credit lines in the borrower's credit file
Months Since Last Delinquency	The number of months since the borrower’s last delinquency
*Borrower Indebtedness*	
Loan Amount to Annual Income	Loan amount to annual income
Annual Instalment to Income	The annual payment owed by the borrower divided by the annual income provided by the borrower during registration
Debt to Income	Borrower's debt to income ratio. Monthly payments on the total debt obligations, excluding mortgage, divided by self-reported monthly income.

The first variable in the Table is a grade, from A to G, assigned by Lending Club to each loan. The grade is a measure for borrower assessment. Each one of the 7 grades has 5 subgrades, so there are 35 subgrades, from A1 down to G5. Lending Club claims that it uses a proprietary credit grading system that looks at borrower credit information and other data provided in the borrower application to assign the grade. The next variable is loan interest rate. Lending Club’s interest rates for each loan grade is the result of the following equation: Lending Club base rate plus adjustment for risk and volatility. In 2015 the subgrade A1 charged an interest rate of 5.32%, and the G5 a 28.99%.

Among the variables measuring loan characteristics, 14 different loan purposes are included, from the most common debt consolidation to wedding loans or loans to start up a small business. Lending Club focuses on personal loans, but it has entered the business loans market. Another variable is loan amount. Borrower characteristics include annual income provided by the borrower during registration, the length of time that workers have been with their current employer and current housing situation, like own, mortgage and rent. Credit history is measured with 7 variables, which assess the length of credit history, the number of inquiries by creditors, or the number of past-due incidences of delinquency in the borrower’s credit file. Finally, to study the role of indebtedness, 3 ratios are included, that relate loan amount, loan annual installment and debt to annual income. Certain loan applicants are required to submit documents that verify the income stated in their loan request.

Tables 2 and 3 show Pearson’s correlation coefficients for continuous variables, and point-biserial correlation coefficients for discrete variables. The latter are the correlation coefficients used when one variable is dichotomous. Results show, as expected, a high correlation between subgrade and interest rate (-0.969). But the rest of correlation coefficients are not high, neither do multicollinearity problems arise. Among the continuous variables, the highest linear relationship is obtained between subgrade and revolving utilization (-0.491). As for discrete variables, the highest correlation coefficient is obtained between subgrade and rented house (-0.124). Results are coherent, because a certain linear relationship is expected between explanatory variables and subgrade. These tables are useful to know which factors better explain the grade assigned by Lending Club linearly, but the relationship could be non-linear [65]. For example, the grade assigned to a retired borrower could be negatively affected if he is living in a rented house, whereas it could be irrelevant for a recently married young couple. Lending Club algorithm is kept secret: the company affirms that the loan grade is the result of a formula that takes into account the applicant’s FICO score, his credit attributes, and other application data too. The FICO score is not built on variables such as annual income, debt-to-income ratio or job stability; its algorithm is also kept secret [30].

**Table 2:** Pearson’s correlation coefficients among continuous explanatory variables (N = 24,449).

	Subgrade	Interest rate	Loan amount	Annual income	Employment Length	Credit History Length	Delinqency 2 Years	Inquiries Last 6 Months	Public Records	Revolving Utilization	Open Accounts	Months Since Last Delinqency	Loan Amount to Annual Income	Annual Instalment to Income	Debt to Income
Subgrade	1	-.969	-.177	-.024	.063	.146	-.177	-.103	-.106	-.491	.021	.127	-.116	-.191	-.090
Interest rate		1	.161	.020	-.067	-.157	.176	.114	.113	.494	-.036	-.130	.109	.190	.092
Loan amount			1	.256	.114	.178	-.032	-.005	-.049	.045	.180	.001	.569	.559	.036
Annual income				1	.116	.174	.020	.024	-.013	.019	.149	-.024	-.240	-.238	-.113
Employment Length					1	.287	.021	-.004	.071	.000	.096	.023	-.052	-.057	.046
Credit History Length						1	.070	.013	.059	-.050	.223	-.004	-.062	-.078	.032
Delinquency 2 Years							1	.001	.007	-.034	.020	-.551	-.058	-.045	-.026
Inquiries Last 6 Months								1	.018	-.071	.100	.010	-.035	-.027	-.001
Public Records									1	.075	-.005	.047	-.043	-.034	.001
Revolving Utilization										1	-.091	.082	.004	.040	.280
Open Accounts											1	.026	-.053	-.058	.286
Months Since Last Delinquency												1	.016	.006	.049
Loan Amount to Annual Income													1	.985	.104
Annual Instalment to Income														1	.108
Debt to Income															1

**Table 3:** Point-biserial correlation coefficients for discrete variables (N = 24,449).

	Subgrade	Interest rate	House: Own	House: Mortgage	House: Rent	House: Other	Wedding	Credit card	Car loan	Major purchase	Home improve-ment	Debt consoled-ation	House	Vacation	Other	Medical	Moving	Renew-able energy	Educational	Small business
Subgrade	1	-.969	.018	.118	-.124	-.018	.004	.031	.086	.072	.072	-.104	.005	.022	.003	.017	.006	.011	-.012	-.087
Interest rate		1	-.014	-.130	.133	.019	.002	-.032	-.089	-.077	-.072	.101	-.005	-.023	.011	-.016	-.001	-.007	.020	.070
House: Own			1	-.247	-.295	-.019	-.011	-.037	.019	.030	.040	-.024	.000	.000	.028	.015	-.013	.007	-.008	-.019
House: Mortgage				1	-.845	-.053	-.034	.015	.006	-.001	.209	-.065	-.016	-.015	-.053	-.007	-.058	.013	-.027	.036
House: Rent					1	-.063	.040	.006	-.016	-.016	-.227	.078	.016	.016	.037	-.002	.066	-.016	.030	-.027
House: Other						1	-.006	-.002	-.005	.003	-.005	-.004	.001	-.007	.003	.007	-.008	-.003	.011	.020
Wedding							1	-.062	-.031	-.042	-.046	-.145	-.015	-.017	-.057	-.022	-.021	-.008	-.018	-.034
Credit card								1	-.074	-.102	-.110	-.347	-.037	-.041	-.137	-.052	-.051	-.020	-.043	-.082
Car loan									1	-.050	-.054	-.172	-.018	-.020	-.068	-.026	-.025	-.010	-.021	-.041
Major purchase										1	-.074	-.236	-.025	-.028	-.093	-.035	-.034	-.013	-.029	-.056
Home improvement											1	-.255	-.027	-.030	-.100	-.038	-.037	-.014	-.032	-.060
Debt consolidation												1	-.086	-.096	-.317	-.121	-.118	-.046	-.100	-.190
House													1	-.010	-.034	-.013	-.013	-.005	-.011	-.020
Vacation														1	-.038	-.014	-.014	-.005	-.012	-.023
Other															1	-.048	-.046	-.018	-.039	-.075
Medical																1	-.018	-.007	-.015	-.029
Moving																	1	-.007	-.015	-.028
Renewable energy																		1	-.006	-.011
Educational																			1	-.024
Small business																				1

Table 4 shows a cross tabulation for discrete variables from the exploratory analysis. A hypotheses test has been included, by means of a Chi-square test. The Chi-square test is used to discover if there is a statistically significant association between two categorical variables. Of the 24,449 loans analyzed, 2,666 are defaulted (10.9%) and 21,783 non-defaulted (89.1%). There is a clear relationship between the grade assigned by Lending Club and the loan status as follows. 94.4% of A-grade loans are fully paid. This percentage gradually lowers down to 61.8% for G-grade loans. Differences are statistically significant (p<0.001). The grade assigned by Lending Club matters and helps to reduce the asymmetric information problem between borrowers and lenders. Loan purpose is a factor that also explains default. For lenders, the less risky loan purpose is wedding loans, with a 92.8% repayment rate. And the most risky is small businesses funding, with a 78.1% repayment rate (p<0.001). This tells us that there is a statistically significant association between small business and default. In fact, the differences were statistically significant in 10 out of 14 loan purposes analyzed. As for the current housing situation, mortgage or own are the less risky, facing rent or other. The differences are statistically significant.

**Table 4:** Exploratory study on discrete variables.

	Loan reimbursed (%)			
Predictors	Yes	No	% (N)	Chi^2^, sig
Grade				
*A*	94.4	5.6	32.3 (7,901)	342.041***
*B*	89.7	10.3	31.7 (7,757)	4.266**
*C*	85.5	14.5	20.2 (4,927)	82.658***
*D*	82.8	17.2	11.6 (2,826)	130.255***
*E*	80.3	19.7	3.2 (785)	65.250***
*F*	74.7	25.3	0.8 (198)	42.300***
*G*	61.8	38.2	0.2 (55)	42.218***
Loan purpose				
*Wedding*	92.8	7.2	2.5 (595)	8.551***
*Credit card*	92.4	7.6	13.0 (3,064)	38.988***
*Car loan*	92.1	7.9	3.5 (831)	7.843***
*Major purchase*	91.6	8.4	6,5 (1,518)	10.863***
*Home improvement*	90.7	9.3	7.4 (1,751)	5.399**
*Debt consolidation*	89.0	11.0	44.6 (10,499)	0.286
*House*	88.4	11.6	0.9 (215)	0.112
*Vacation*	88.3	11.7	1.1 (264)	0.187
*Other*	87.6	12.4	11.1 (2614)	6.940***
*Medical*	85.7	14.3	1.8 (420)	4.987**
*Moving*	85.2	14.8	1.7 (399)	6.247**
*Renewable energy*	85.2	14.8	0.3 (61)	0.925
*Educational*	83.6	16.4	1.2 (287)	8.900***
*Small business*	78.1	21.9	4.3 (1,012)	132.010***
Housing situation				
*Mortgage*	90.1	9.9	41.4 (10,121)	16.881***
*Own*	89.2	10.8	7.9 (1,940)	0.014
*Rent*	88.3	11.7	50.3 (12,290)	14.835***
*Other*	82.5	17.5	0.4 (97)	4.395**

Number of loans analyzed: 24,449. Defaulted: 2,666 (10.9%). Non-defaulted: 21,783 (89.1%).

*** significant at the 1% level

** significant at 5% the level.

Table 5 shows the exploratory study on the continuous variables. The mean and the standard deviation are disclosed in all the cases: defaulted and non-defaulted. As expected, the interest rate is a relevant variable: defaulted loans paid, on average, 12.3%, a higher interest rate than non-defaulted loans, a 10.8%. The independent-samples t-test compares the means between two groups in the same continuous, dependent variable. Differences in interest rates are statistically significant (p<0.001), although the difference is just 1.5 points. Among Lending Club borrowers (N = 24,449), considering their annual income, there was a statistically significant difference between the defaulted group (mean = $59,595) and the non-defaulted group (mean = $68,391). Therefore, there are also statistically significant differences in annual income (p<0.001). Considering the length of employment, there was no statistically significant difference between the defaulted group (mean = 4.60 years) and the non-defaulted group (mean = 4.68) (p ≥ 0.05). In other words, we fail to reject the null hypothesis that there is no difference in employment length between defaulted and non-defaulted loans.

**Table 5:** Exploratory study on continuous variables.

Predictors	All (N = 24,449)		Failed (N = 2,666)		Non-failed (N = 21,783)		T-test, sig
	Mean	St dev	Mean	St dev	Mean	St dev	
*Borrower Assessment*							
Interest Rate	0.110	0.032	0.123	0.030	0.108	0.031	24.342***
*Loan Characteristics*							
Loan Amount	9,499	6,253	9,385	6,420	9,513	6,232	-0.997
*Borrower Characteristics*							
Annual Income	67,432	66,843	59,595	46,632	68,391	68,850	-8.653***
Employment Length	4.67	3.53	4.60	3.55	4.68	3.53	-1.076
*Credit History*							
Credit History Length	6,483	2,497	6,323	2,488	6,503	2,497	-3.439***
Delinquency 2 Years	0.15	0.49	0.18	0.51	0.14	0.48	3.251***
Inquiries Last 6 Months	0.85	1.06	1.07	1.18	0.82	1.04	10.251***
Public Records	0.0566	0.24	0.0911	0.298	0.0524	0.235	6.326***
Revolving Utilization	0.46	0.28	0.53	0.284	0.45	0.284	13.002***
Open Accounts	9.13	4.40	8.92	4.63	9.15	4.42	-2.516**
Months Since Last Delinquency	33.64	22.40	32.96	22.42	33.74	22.39	-1.018
*Borrower Indebtedness*							
Loan Amount to Annual Income	0.166	0.10	0.183	0.12	0.163	0.10	8.492***
Annual Instalment to Income	0.064	0.041	0.072	0.046	0.063	0.040	9.842***
Debt to Income	12.86	6.68	13.48	6.66	12.78	6.68	5.007***

Number of loans analyzed: 24,449. Defaulted: 2,666 (10.9%). Non-defaulted: 21,783 (89.1%).

*** significant at the 1% level

** significant at 5% the level.

All credit history variables present differences in the expected sign, and all of them are statistically significant, except for the number of months since the borrower’s last delinquency. The three variables measuring borrower indebtedness present statistically significant differences: the higher the indebtedness or the loan payments to income ratio, the higher the probability of default is.

To sum up, within the Lending Club data analyzed, the hypotheses are partially accepted: the higher the interest rate, the higher the default probability is. Loan characteristics, such as loan purpose; borrower characteristics, such as annual income and current housing situation; credit history and borrower indebtedness do matter. However, variables such as loan amount or the length of employment do not seem to be relevant within the data analyzed.

The main techniques to develop the probability of default are classification models and survival analysis, which facilitate estimating not only whether but also when a customer defaults [65]. The logistic regression is a well-established technique employed in evaluating the probability of occurrence of a default [70] but recent research in credit scoring emphasizes the importance of not only distinguishing ‘good’ and ‘bad’ borrowers, but also predicting when a customer will default [56], [71], [72]. We have performed a survival analysis and a logistic regression analysis. Both techniques use the same data and the same explanatory variables, but the dependent variable differs. In logistic regression, the dependent variable is binary or dichotomous (e.g., default or non-default). By contrast, in the survival analysis the dependent variable is the time until the occurrence of an event of interest; in other words, the dependent variable is how long the loan has survived. This is done by means of Cox regression, which relates survival time and explanatory variables.

Table 6 shows the survival analysis results, by means of 33 Cox regressions, one for each explanatory variable. The Table provides the regression coefficients, standard errors, risk ratios and significance of p-values. The regression coefficient is interpreted as a k-fold increase in risk. Hence, a positive regression coefficient for an explanatory variable means that the risk is higher. Risk ratio can be interpreted as the predicted change in the risk for a unit increase in the explanatory variable. The Table reveals important practical findings for lenders. For example, by comparing loan purposes, the riskiest is ‘small business’ and the least risky is ‘wedding purpose’. The risk of loans for ‘small business’, ceteris paribus, is 2.279 times higher than the risk of loans for ‘no small business’. By contrast, the risk of ‘wedding’ loans is 0.647 times lower than ‘no wedding’ loans. The significance test for the coefficient tests the null hypothesis that it equals zero. In both small business loans and wedding loans, statistically significant differences have been found (p<0.000). Results are coherent with the explanatory analysis, but more precise.

**Table 6:** Cox regression analysis for loans’ survival time.

Predictors	Parameter estimate	Standard error	Risk ratio
*Loan purpose*			
Wedding	-0.435***	0.154	0.647
Credit card	-0.423***	0.069	0.655
Car loan	-0.353***	0.125	0.702
Major purchase	-0.300***	0.091	0.741
Home improvement	-0.193**	0.081	0.825
Debt consolidation	0.021	0.040	1.021
House	0.073	0.201	1.076
Vacation	0.074	0.181	1.076
Other	0.160***	0.059	1.173
Medical	0.299**	0.131	1.348
Moving	0.329**	0.132	1.390
Renewable energy	0.339	0.334	1.403
Educational	0.443***	0.147	1.557
Small business	0.824***	0.070	2.279
*Housing situation*			
Mortgage	-0.176***	0.041	0.838
Own	-0.007	0.073	0.993
Rent	0.161***	0.040	1.175
Other	0.492*	0.251	1.635
*Borrower Assessment*			
Subgrade	-0.071***	0.003	0.931
Interest rate	14.444***	0.619	1873887
*Loan Characteristics*			
Loan Amount	0.000	0.000	1.000
*Borrower Characteristics*			
Annual Income	0.000***	0.000	1.000
Employment Length	-0.006	0.006	0.994
*Credit History*			
Credit History Length	0.000***	0.000	1.000
Delinquency 2 Years	0.120***	0.034	1.128
Inquiries Last 6 Months	0.186***	0.016	1.204
Public Records	0.470***	0.061	1.600
Revolving Utilization	0.925***	0.070	2.522
Open Accounts	-0.012**	0.005	0.988
Months Since Last Delinquency	-0.002	0.001	0.988
*Borrower Indebtedness*			
Loan Amount to Annual Income	1.578***	0.174	4.845
Annual Instalment to Income	4.654***	0.436	104.982
Debt to Income	0.015***	0.003	1.015

Number of loans analyzed: 24,449. Defaulted: 2,666 (10.9%). Non-defaulted: 21,783 (89.1%).

*** significant at the 1% level

** significant at 5% the level

* significant at the 10% level.

**Fig 1:**
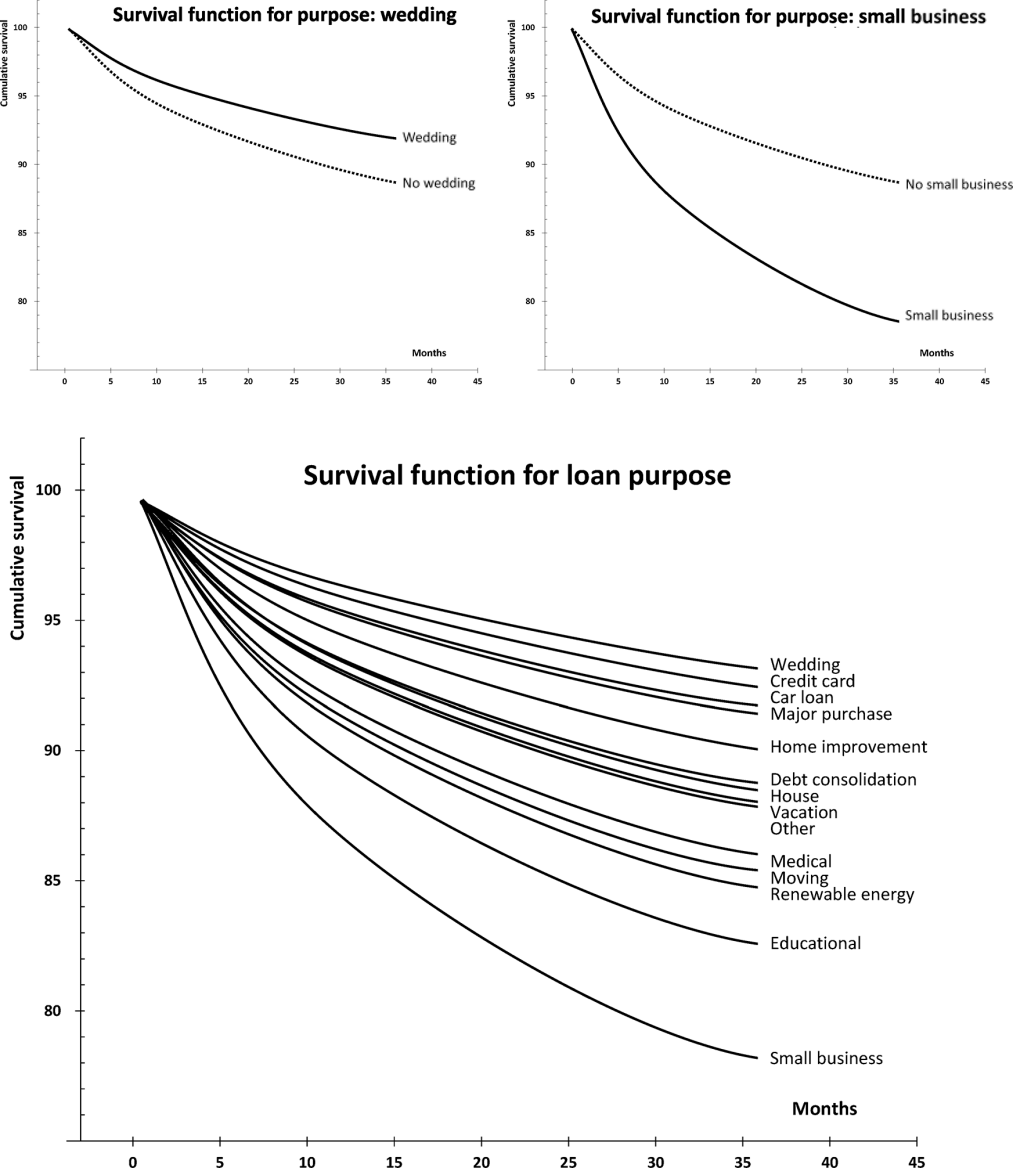
Relationship between survival functions for the Cox model.

Survival curves can be useful for lenders, because they show the probabilities of default at a certain point of time (Fig 1). The chart at the bottom displays the survival curves for each loan purpose. The chart at the top left displays the survival curves for ‘wedding’ loans. It can be clearly appreciated that the probability of survival is higher for ‘wedding’ purposes than for ‘non-wedding’ purposes. The chart at the top right displays the survival curves for ‘small business’ loans. Here, the probability of survival is lower for ‘small business’ purposes than for ‘no small business” purposes.

With the aim of analyzing the predictive capability of the variables, 7 logistic regression models have been performed. In classification and prediction studies a common practice is to separate into primary sample (train sample) and test sample (holdout sample). Lau [73] criticizes some of the early studies because holdout samples were drawn from the same time period as the original samples, lacking intertemporal validation and moreover, this is not a real-world situation [74]. This practice has long been recognized as generating over-optimistic inference but practitioners frequently do little to address it [75]. This is not our case. Algorithms were trained from the point of view of a financial analysts situated on the 1^st^ of July 2011. At that time, the analyst had 137 defaulted loans of 2008 first semester available, all of them 36 month loans. Defaulted loans were matched with 137 non-defaulted loans. The paired matched sample technique is commonly used in this kind of studies [70], [76]. So the primary sample contains 274 loans. The holdout sample contains all the loans funded through Lending Club in 2011 third trimester, from the 1^st^ of July 2011 to the 30^th^ of September 2011. These are 3,788 loans of 36 months length. Therefore, the analyst could know their status on the 30^th^ of September 2014. By analyzing the status of the holdout sample loans, 401 are defaulted and 3,387 are non-defaulted. Then, the accuracy of each of the 7 models can be calculated, measuring the percentage of correctly classified loans.

Table 7 shows the performance of the 7 logistic regression models. Model 1 uses only the subgrade as an explicative variable; further models add variables up to model 7, a full model containing all the explicative variables. Logistic regression provides several statistics that indicate the significance of each variable and some goodness-of-fit measures by means of the Hosmer–Lemeshow test and the Nagelkerke-statistics. The Hosmer–Lemeshow test is a statistical test based on grouping cases into deciles of risk and comparing the observed probability with the expected probability within each decile. The p-value in Table 7 is above 0.05, which implies that the proposed model fits the data well. In ordinary linear regression, the primary measure of model fit is R-square, which is an indicator of the percentage of variance in the dependent variable explained by the model. But the R-square measure is only appropriate to linear regression. The Nagelkerke-statistic is just a normalized version of the R-square computed from the likelihood ratio used in a logistic regression [77]. Furthermore, Table 7 shows the total percentages of correctly predicted cases for each model both in the primary sample as well as in the holdout sample.

**Table 7:** Logistic regression analysis for potentially insolvent status of P2P borrowers, showing B coefficients and significance levels.

	Model 1	Model 2	Model 3	Model 4	Model 5	Model 6	Model 7
*Borrower Assessment*							
Subgrade	0.086***		0.095***	0.089***	0.087***	0.090***	-0.008
Interest Rate		-26.102***					-32.924***
*Purpose*							
Car			-2.742*				-2.383
Credit Card			-1.717				-1.605
Debt Consolidation			-1.731				-1.527
Educational			-2.113				-1.646
Home Improvement			-1.588				-1.416
House			-2.150				-1.980
Major Purchase			-2.555*				-2.265
Medical			-2.708				-2.264
Moving			19.148				19.461
Small Business			-1.878				-1.372
Other			-1.957*				-1.966
*Borrower Characteristics*							
Housing Situation: Own				-20.784			-20.826
Housing Situation: Mortgage				-20.592			-20.327
Housing Situation: Rent				-20.498			-20.334
Housing Situation: Other				-20.843			-20.609
Annual Income				0.001			0.001
*Credit history*							
Inquiries Last 6 Months					-0.327***		-0.337**
Delinquency 2 Years					0.357		0.438
Public Records					0.413		0.428
Revolving Utilization					-0.331		-0.430
*Indebtedness*							
Loan Amount to Annual Income						-11.227**	-11.685**
Annual Instalment to Income						24.219**	24.172**
Hosmer–Lemeshow test	0.730	0.942	0.449	0.766	0.236	0.168	0.505
Nagelkerke R Square	0.076	0.078	0.114	0.088	0.128	0.124	0.212
Correctly predicted (primary sample)	58.8%	58.0%	59.7%	60.2%	60.1%	62.0%	64.6%
Correctly predicted (holdout sample)	75.2%	62.0%	72.8%	76.0%	71.9%	80.6%	65.1%

Primary sample comprises 274 loans funded in 2008 first semester, where 137 are defaulted and 137 non-defaulted. Test sample comprises all the 3,788 loans funded in 2011 third trimester, where 401 are defaulted and 3,387 are non-defaulted.

*** significant at the 1% level

** significant at 5% the level

* significant at the 10% level.

In model 1, where the subgrade is the independent variable, the total percentage of correctly predicted cases is 58.8% for the primary sample and 75.2% for the holdout sample. It is worth pointing out that the prediction is better in the test than in the train; this is an example of underfitting. A possible explanation lies in the economies of learning, because Lending Club’s loans in 2008 were issued under an embryonic credit model. Another reason is that those loans happened during the 2008 economic crisis and many loans apparently non-risky finally defaulted. The contrary situation, known as overfitting, is more common [78]. Overfitting generally arises when a model has too many parameters relative to the number of observations. An overfitted model will generally have a poor predictive performance, because it can exaggerate minor fluctuations in the data [70].

It must be remembered that the Pearson correlation coefficient between interest rate and subgrade is -0.969, very close to 1, given the close relationship between both variables. Model 2 uses the interest rate, and its accuracy is not improved, neither in the primary sample nor in the test sample. By including purpose variables (model 3) accuracy does not improve either. Model 4, incorporating variables on borrower characteristics, such as current housing situation and loan amount, hardly improves its accuracy. The same happens with model 5, including credit history variables. This can be interpreted by the role of subgrade, which incorporates most of the variables predicting default. It must be highlighted that correlation is a linear relationship; and the relationship between grade and variables could be more complex. Model 6 brings a clear improvement, including indebtedness variables. Here, the correctly predicted cases in the primary sample increases from 58.8% to 62% and the correctly predicted percentage in the holdout sample increases from 75.2% to 80.6%. Finally, the full model improves the classification accuracy in the primary sample (from 62% to 64.6%), but lowers the prediction accuracy in the holdout sample (from 80.6% to 65.1%). It is an overfitted model, since the train sample is well adjusted, but it fails in the test.

To sum up, the subgrade assigned by the P2P lending site, based on FICO credit score and other attributes, is the most important variable and, in the sample data used, reduces the information asymmetry suffered by the lender, which is one of the main problems in this business model. But the use of mathematical models (means test, logistic regression and survival analysis) can improve loan selection by individual investors. This is not a big surprise, but many lenders pay attention to aspects that have not turned out to be related to the probability of default [79], [80]. Ravina [79] has studied the effect of personal characteristics in P2P lending sites, finding that beauty, race, age, and other personal characteristics are taken into account by lenders. Beautiful applicants have higher probability of getting loans, pay less, but have similar default rates. Pope and Sydnor [81] find evidence of significant racial disparities in P2P lending. Gonzalez and Loureiro [37] study the effect of photographs in lending, finding that gender, perceived age and attractiveness of borrowers affect lenders’ decisions. Mild, Waitz and Wöckl [80] find that lenders fail to transform the available information into right decisions. Lin, Prabhala and Viswanathan [19] find that friendships of borrowers act as signals of creditworthiness, increasing thus the probability of successful funding. Duarte, Siegel and Young [82] find that borrowers who appear more trustworthy have higher probabilities of having their loans funded. Behavioral finance, a discipline that combines Psychology and Finance, tries to explain financial markets’ evidence of irrationality [83] and also is used to explain P2P credit markets. Zhang and Liu [39] find evidence of herding in P2P lending: lenders infer the creditworthiness of borrowers by observing peer lending decisions and use publicly observable borrower characteristics to moderate their inferences. Yum, Lee and Chae [5] also find herding behavior although they could not test the repayment performance implications since most of the loans have not matured. P2P lenders should take into account the variables that matter, avoiding the error of judgement, avoiding irrelevant variables and irrational herding. Future research into this topic could include the study of the non-linear relationship among variables and its association with the probability of default.

## Conclusions

P2P lending companies may bear less transaction costs than conventional financial institutions do, since its business model is simpler: they do not capture deposits, they are not under strict banking regulations, they do not maintain idle balances; they just put borrowers in contact with lenders. Besides, this is done by means of an online platform where most of the processes are automatized. Operating cost is the most important factor explaining interest margins in banking, and P2P lending platforms–like other online businesses- have the use of technologies as strength. This can lead to improving the efficiency, a very important factor in a market where money is bought and sold. Money is a non-differentiated product and its price, the interest rate, is what matters most. P2P lending can alleviate credit rationing, especially for those borrowers placed in the long tail of credit. These advantages could explain P2P lending growth, but it is not problem-free. In the banking business model, the credit risk is assumed by the financial institution, which has risk management departments with skilled financial analysts, supposedly more expert than individual lenders. In fact, in some countries and US states, the amount of money an individual lender can invest per platform is limited by law, or even forbidden. In the P2P lending business model, the credit risk is assumed by individuals, who put at risk their money lending to other individuals. The information asymmetry problem is huge. For this reason, it is important for the P2P lending site to offer quality information about the loan. This information can be provided by third parties, such as external credit scores, or it can be extracted from the platform itself, such as the grade assigned to each loan.

The paper analyzes whether the information provided by the P2P lending site, a grade that qualifies the loan, complemented with loan and borrower characteristics, explains loan defaults and reduces information asymmetry. Firstly, a hypotheses test and a survival analysis have been performed on the factors explaining loan defaults. Secondly, a regression logistic model has been proposed to predict loan default. The empirical study uses data from Lending Club, the biggest US P2P lending site. To assure intertemporal validation, data contains a primary sample with 274 loans funded in 2008 first semester and a test sample with all the 3,788 loans funded by Lending Club in 2011 third trimester. These are 36 month loans, so its final status (401 defaulted and 3,387 non-defaulted) was known the 30^th^ September 2014.

The study results show that there is a clear relationship between the grade assigned by Lending Club and the probability of default. 94.4% of A-grade loans were reimbursed. This percentage gradually decreases to 61.8% for G-grade loans. The interest rate assigned depends on the grade assigned and the higher the interest rate, the higher the default probability is. Loan purpose is also a factor explaining default: wedding is the less risky loan purpose and small business is the riskiest. Borrower characteristics, such as annual income, current housing situation, credit history, and borrower indebtedness are relevant variables. No statistically significant differences are found in loan amount or length of employment. The regression model shows that the grade assigned by Lending Club is the variable with the highest predictive capability. Total percentages of correctly predicted loans range from 58% to 64.4% in the primary sample, and from 62% to 80.6% in the holdout sample. Although there are studies analyzing the accuracy of credit scores such as FICO, like Fuller and Dawson [84], it is difficult to establish comparisons, because they refer to different periods.

To sum up, Lending Club, like other P2P lending sites, discloses all the historic information on loans funded, qualified by a loan grade, what mitigates information asymmetry. Some lenders may take into account irrelevant aspects when deciding to lend, as shown in the research literature [79], [80]. We encourage the use of sound credit scoring models, rooted in statistical techniques, based on robust data, thus avoiding the error of judgment.
